# The Effect of *Trichoderma harzianum* Hypovirus 1 (ThHV1) and Its Defective RNA ThHV1-S on the Antifungal Activity and Metabolome of *Trichoderma koningiopsis* T-51

**DOI:** 10.3390/jof9020175

**Published:** 2023-01-28

**Authors:** Jiaqi You, Zheng Hu, Chaohan Li, Hongjuan Yang, Lihua Zhu, Biting Cao, Ronghao Song, Weihong Gu

**Affiliations:** 1 Horticultural Research Institute, Shanghai Academy of Agricultural Sciences, Shanghai Key Lab of Protected Horticultural Technology, Shanghai 201106, China; 2 Institute for Agri-Food Standards and Testing Technology, Shanghai Academy of Agricultural Science, Shanghai 201106, China; 3 Plant Protection Research Institute, Shanghai Academy of Agricultural Science, Shanghai 201106, China

**Keywords:** *Trichoderma*, mycovirus, antifungal activity, metabolome, biological control

## Abstract

Mycoviruses widely exist in filamentous fungi and sometimes cause phenotypic changes in hosts. *Trichoderma harzianum* hypovirus 1 (ThHV1) and its defective RNA ThHV1-S were found in *T. harzianum* and exhibited high transmissibility. In our previous study, ThHV1 and ThHV1-S were transferred to an excellent biological control agent *T. koningiopsis* T-51 to form a derivative strain 51-13. In this study, we assessed the metabolic changes in strain 51-13 and antifungal activity of its culture filtrate (CF) and volatile organic compounds (VOCs). The antifungal activity of CF and VOCs of T-51 and 51-13 was different. Compared with the CF of T-51, that of 51-13 exhibited high inhibitory activity against *B. cinerea*, *Sclerotinia sclerotiorum*, and *Stagonosporopsis cucurbitacearum* but low inhibitory activity against *Leptosphaeria biglobosa* and *Villosiclava virens*. The VOCs of 51-13 exhibited high inhibitory activity against *F. oxysporum* but low inhibitory activity against *B. cinerea*. The transcriptomes of T-51 and 51-13 were compared; 5531 differentially expressed genes (DEGs) were identified in 51-13 with 2904 up- and 2627 downregulated genes. In KEGG enrichment analysis, 1127 DEGs related to metabolic pathways (57.53%) and 396 DEGs related to biosynthesis of secondary metabolites (20.21%) were clearly enriched. From the CF of T-51 and 51-13, 134 differential secondary metabolites (DSMs) were detected between T-51 and 51-13 with 39 up- and 95 downregulated metabolites. From these, 13 upregulated metabolites were selected to test their antifungal activity against *B. cinerea*. Among them, indole-3-lactic acid and p-coumaric acid methyl ester (MeCA) exhibited strong antifungal activity. The IC_50_ of MeCA was 657.35 μM and four genes possibly related to the synthesis of MeCA exhibited higher expression in 51-13 than in T-51. This study revealed the mechanism underlying the increase in antifungal activity of T-51 because of the mycovirus and provided novel insights in fungal engineering to obtain bioactive metabolites via mycoviruses.

## 1. Introduction

Mycovirus is a type of virus that can infect and replicate in filamentous fungi, yeasts, and oomycetes [1]. Mycoviruses have double- or single-stranded RNA, or circular single-stranded DNA as the genome [2]. Mycoviruses widely exist in fungi but often do not affect the host [1]. However, some mycoviruses’ infections change the biological characteristics of their host. Mycoviruses are getting increasing attention for their role in the pathogenesis of fungal infections in plants, insects, and humans. Particularly, the mycoviruses causing the hypovirulence phenotype of plant pathogenic fungi are being studied in detail because of their potential to be a biological control agent [2]. However, the roles of mycoviruses in biological control fungus are rarely reported.

Hypovirulence-causing mycoviruses in the pathogenic fungi of plants, such as Cryphonectria hypovirus 1 (CHV1), which reduced the infection of *Cryphonectria parasitica* in chestnut, have been used for the biological control of pathogenic fungi of plants [3]. *Sclerotinia sclerotiorum* hypovirulence-associated DNA virus 1 (SsHADV-1), a ssDNA mycovirus, induced hypovirulence in its host; it could be transmitted through an insect vector and helped to control *S. sclerotiorum* infection in rapeseed [4]. *Botrytis cinerea* hypovirus 1(BcHV1) attenuated the virulence of its host by inhibiting the infection cushion formation [5]. 

Moreover, mycoviruses may affect the secondary metabolites of their host fungi. Compared with the volatile organic compounds (VOCs) produced by the virulent *B. cinerea* isolate, the VOCs produced by *B. cinerea* isolate QT5-19, which contains a hypovirulence-causing mycovirus *B. cinerea* partitivirus 2 (BcPV2), exhibited antifungal activity against *B. cinerea* and *S. sclerotiorum*, and promoted tomato plant growth [6,7]. The well-known killer yeast strains, such as *Saccharomyces cerevisiae*, can kill other yeasts by producing killer toxin, which are cytoplasmically inherited killer characteristics as two high-weight dsRNAs that do not follow Mendel’s laws of inheritance [8]. The L-A double-stranded RNA virus (4.6 kb) [9] and its satellite RNA M (1.5–2.4 kb) [10] were characterized in different killer yeasts; the different killer toxins were encoded by M-dsRNA and maintained by a helper virus L-A. Satellite RNA M and their analyzed helper virus sequences showed co-evolution [11] and the killer viruses can be transferred between various yeast species. A laboratory constructed “superkiller” yeast strain demonstrated that the same L-A can help different M components [11]. In filamentous fungi, some mycoviruses affect the toxin metabolite of their host. For example, high titer of *Alternaria alternata* chrysovirus 1 (AaCV1) enhanced the pathogenicity of its host by increasing the level of the AK-toxin by 13-fold, which is essential for the pathogenicity of the *A. alternata* Japanese pear pathotype [12]. *Aspergillus ovhraceus* virus (AoV) increased the production of ochratoxin A (OTA), which is an important contaminant in food and feed commodities [13]. 

*Trichoderma* spp. are fungi well-known for the biological control of other fungi due to their ability to produce a wide range of antibiotics and cell wall-degrading enzymes that enable them to parasitize other fungi [14]. More than 180 antibiotics have been isolated from *Trichoderma* spp. [15], including nonvolatile and volatile compounds. In addition, some secondary metabolites exhibit growth-promoting and systemic-defense-inducing activities in plants [16].

*T. koningiopsis* T-51 is an excellent biological control agent [17,18]. It effectively controlled tomato gray mold disease (caused by *B. cinerea*) and watermelon fusarium wilt (caused by *Fusarium oxysporum*) and exhibited plant-growth-promoting ability in tomato and watermelon. In our previous study, we reported a mycovirus from *T. harzianum* (ThHV1) and its defective RNA (ThHV1-S), and demonstrated that ThHV1-S decreased mycoparasitism and increased the antifungal activity of the culture filtrate (CF) of the host [19]. A derivative strain 51-13 was obtained by transmission of ThHV-1 and ThHV1-S into the wild-type strain of *T. koningiopsis* T-51. The derivative strain showed increased antifungal activity against *B. cinerea* [19]. In this study, the antifungal activity of CF and VOCs produced by strain 51-13 were evaluated against several pathogenic fungi of plants and the mechanism underlying the effect of ThHV1-S on the host’s antifungal activity was explored.

## 2. Materials and Methods

### 2.1. Fungal Isolates

*T. koningiopsis* T-51 was isolated from soil in Hubei province, China and is a biological control agent against *B. cinerea* that causes gray mold disease in tomato and other plants. *T. koningiopsis* strain 51-13 is a derivative of *T. koningiopsis* T-51, carrying the mycovirus ThHV1 and its defective RNA, ThHV1-S [19]. Six pathogenic fungal isolates from plants including *B. cinerea* B05.10, *Sclerotinia sclerotiorum* Ss1880 and *Leptosphaeria biglobosa* GNXH1-26 from oil rape, *Stagonosporopsis cucurbitacearum* Sc2021 and *F. oxysporum* Fon2019 from watermelon, and *Villosiclava virens* HWD-2 from rice were used in this study. All fungi were stored in 20% glycerol solution at −80 °C and grown on potato dextrose agar (PDA) media at 20 °C.

### 2.2. Scanning Electron Microscopic Observation of Fungal Morphology

The mycelial morphology of T-51 and 51-13 was observed using a scanning electron microscope (SEM). PDA media was melted and cooled to approximately 50 °C. A sterilized cover glass (1 × 1 cm) was dipped in warm PDA medium and placed on a PDA plate. A membrane of the medium was formed on the cover glass when the medium solidified. T-51 or 51-13 was inoculated on the medium membrane on the cover glass and incubated at 25 °C for 48 h. The mycelia with the membrane of the medium were peeled off from the cover glass and immediately fixed in 2.5% glutaral at 4 °C overnight, washed in phosphoric acid buffer (0.1 M, pH 7.0) 3 times, fixed in 1% osmic acid solution for 1–2 h, and rinsed in phosphoric acid buffer (0.1 M, pH 7.0) 3 times. The sample was dehydrated, the sample with ethanol solution of gradient concentration (30%, 50%, 70%, 80%, 90%, 95%, and 100%), and further treated overnight with pure isoamyl acetate. The samples were critical point dried, coated with gold, and examined using an SEM (SU8010, Hitachi, Tokyo, Japan); the conditions were: Vacc = 3kV, Mag = x3.00k, WD = 8.6 mm.

### 2.3. Genomic DNA Sequencing 

The genomic DNA of T-51 was sequenced by Personal Biotechnology Company (Shanghai, China). Briefly, genomic DNA was extracted from T-51 mycelia using the CTAB method. After checking quality and integrity, sequencing libraries were generated using the TruSeq DNA Sample Preparation Kit (Illumina, San Diego, CA, USA) and the Template Prep Kit (Pacific Biosciences, Menlo Park, CA, USA). The genome sequencing was performed using the Pacific Biosciences platform and Illumina Novaseq platform. For variant analysis, 51-13 was resequenced. The extracted DNA was used to construct a library with 400-bp insert. Further, the sequencing data were mapped to the genome sequence of T-51 to identify single nucleotide polymorphisms (SNPs), insertions, and deletions (indels).

### 2.4. Antifungal Activity of CF of Trichoderma Isolates

T-51 and 51-13 were cultured on PDA plates for 2 days at 20 °C in light. Three mycelial plugs (diameter 5 mm) from the edge of colonies of T-51 or 51-13 were inoculated in 100 mL of PDB in a 250-mL glass flask and shaken at 150 rpm and 25 °C for 7 days. The fungus was removed from the CF by successively filtering through a filter paper and microporous membrane with 0.22-µm aperture. The antifungal activity of the *Trichoderma* CF was evaluated using 10% (*v*/*v*) CF with PDA, as described in a previous study [17]. In brief, 2 mL of CF was mixed with 18 mL PDA and poured into a plate with diameter of 9 cm. Mycelial plug of the test pathogenic fungus was inoculated at the center of the plate after the medium solidified. Control consisted of 2 mL of PDB instead of the CF. The plate was kept in an incubator at 20 °C in light. The diameter of fungal colonies was measured daily until the control colony grew to the edge of plate. Each treatment and control group had four plates, and the experiment was repeated twice. The inhibition percentage of the pathogenic fungal growth by CF was calculated using the following formula:

Inhibition percentage = (daily growth rate of control − daily growth rate of treatment)/daily growth rate of control × 100%.

The mycelial morphological characteristics of *B. cinerea* treated with *Trichoderma* CF were observed using an SEM, according to the method described above, using a CF + PDA medium membrane, and PDA as the control. *B. cinerea* was inoculated and grown at 20 °C for 48 h before fixing.

### 2.5. Antifungal Activity of the VOCs Produced by T-51 and 51-13

The antifungal activity of the VOCs of T-51 and 51-13 was assessed using a double dish method, as previously described [20]. Before double dish culture, T-51 and 51-13 were grown on PDA in a 9 cm diameter glass petri dish for 48 h. A PDA plate inoculated with either *B. cinerea* or *F. oxysporum* was placed facing over the plate of T-51 or 51-13 and wrapped with parafilm. A clean PDA plate set double dish culture with the *B. cinerea or F. oxysporum* was used as the control. The double dish sets were incubated at 20 °C and the colony diameter of the pathogenic fungus was measured daily until the control colony grew to the edge of plate. Each treatment and control group had four plates and the experiment was repeated twice. The inhibition percentage of the pathogenic fungal growth was calculated as described in Section 2.4. The mycelial morphology of *B. cinerea* or *F. oxysporum* was observed using SEM, as previously described [18].

### 2.6. Transcription Profiling of T-51 and 51-13

T-51 and 51-13 were cultured in the PDB for 7 days with 3 replicates. For each replication, 1 g of mycelia was collected using a filter paper and immediately frozen in liquid nitrogen for RNA extraction. The CF from the PDB was collected (as described in Section 2.4) and used as the source for the analysis of secondary metabolites. Both transcriptome sequencing and metabolome detection were processed by Wuhan Metware Biotechnology Co., Ltd. (Wuhan, China). The mycelial RNA was extracted using TRizol^®^ reagent (Invitrogen Corporation, Carlsbad, CA, USA). After quality testing, the RNA was sequenced using an Illumina HiSeq^TM^2000. The Trinity method was used for the transcriptome assembly [21] as *T. koningiopsis* did not have a reference genome. Differentially expressed genes (DEGs) from T-51 and 51-13 were screened based on the fold change (FC) ≥ 2 and false discovery rate (FDR) < 0.05. Gene function was annotated based on the databases, including Nr (NCBI non-redundant protein sequences), Pfam (Protein family), KOG/COG (Clusters of Orthologous Groups of proteins), Swiss-Prot (A manually annotated and reviewed protein sequence database), KEGG (Kyoto Encyclopedia of Genes and Genomes), and GO (Gene Ontology). The pathway enrichment of the DEGs was performed based on the KEGG database. 

### 2.7. Analysis of Secondary Metabolites in T-51 and 51-13

For the secondary metabolites’ detection, 10 mL of CF of T-51 or 51-13 was put in a 15-mL tube and immediately frozen in liquid nitrogen. In addition, 10 mL PDB media was prepared as a blank for the analysis of secondary metabolites. The CF samples were sent to Wuhan Metware Biotechnology Co., Ltd. (Wuhan, China) for the detection of secondary metabolites. The samples were extracted using 70% methanal with DMSO (as the internal standard), filtered with a microporous membrane (0.22 μm), and analyzed using ultra-performance liquid chromatography (UPLC, SHIMADZU Nexera X2)-tandem mass spectrometry (MS/MS, Applied Biosystems 4500 QTRAP). The analytical conditions were as follows [22]: HPLC column, Agilent SB-C18 1.8 µm, 2.1 mm × 100 mm; solvent system, water (0.1% formic acid): acetonitrile (0.1% formic acid); gradient program, 95:5 *v*/*v* at 0 min, 5:95 *v*/*v* at 9.0 min, 5:95 *v*/*v* at 10.0 min, 95:5 *v*/*v* at 11.1 min, 95:5 *v*/*v* at 14.0 min; flow rate, 0.35 mL/min; temperature, 40 °C; and injection volume: 4 μL. The effluent was connected to an electrospray ionization (ESI)-triple Q-TRAP-MS. The ESI source operation parameters were as follows: ion source turbo spray; source temperature 550 °C; ion spray voltage (IS) 5500 V (positive ion mode)/−4500 V (negative ion mode); ion source gas I (GSI), gas II (GSII), and curtain gas (CUR) set at 55, 60, and 25.0 psi, respectively; and high collision gas (CAD). Instrument tuning and mass calibration were performed with 10 and 100 μmol/L polypropylene glycol solutions in QQQ and LIT modes, respectively. The QQQ scans were acquired as multiple reaction monitoring (MRM) experiments with the collision gas (nitrogen) set to medium. Qualitative analysis was conducted by comparison of the accurate precursor ions (Q1), product ion (Q3) values, and the retention time (RT), based on the self-built database MWDB (Metware Biotechnology Co., Ltd., Wuhan, China). After obtaining the metabolite MS data of various samples, peak area integration was performed. Finally, the chromatographic peak area was used to determine the relative metabolite contents. The differential secondary metabolites (DSMs) between T-51 and 51-13 were determined by absolute log_2_FC ≥ 1 and variable importance in projection (VIP) ≥ 1., calculated from orthogonal partial least squares-discriminant analysis (OPLS-DA), using R package MetaboAnalystR.

### 2.8. Screening of Candidates Producing Antifungal Metabolites

Candidate antifungal metabolites attributed to the presence of ThHV1-S were selected from the upregulated DMSs when comparing 51-13 to T-51. Furthermore, the compounds present in PDB and downregulated in T-51 compared with PDB were removed from the list of candidates. These DSMs were considered to be associated with the different media consumption ability between the two strains. Overall, 13 commercially available compounds of the screening upregulated DSMs were bought from Shanghai Yuanye Bio-Technology Co., Ltd. and numbered from A to M for analysis. These 13 compounds were used to assess the antifungal activity. Overall, 20 mL of warm PDA media with each compound (1 mM final concentration) was poured into a 9-cm petri dish and a *B. cinerea* mycelial plug of 5-mm diameter (from the edge of a 48-h-old colony) was inoculated at the center of a PDA plate. The PDA plate inoculated with *B. cinerea* was used as a control. Each treatment and control contained 5 replicates. The inhibition of *B. cinerea* growth was measured as described in Section 2.4.

According to the result, various concentrations of compound E (p-coumaric acid methyl ester, MeCA; 800, 600, 400, 200, 100, 80, 10, and 1 μM and 100 and 10 nM) were further tested for antifungal activity, and the IC_50_ values were calculated according to the mycelial growth inhibition curve of *B. cinerea* using program OriginPro 8.0 (OriginLab, MA, USA).

### 2.9. Real-Time Quantitative PCR (qRT-PCR)

According to the results of this study, 4 upregulated DSMs that were possibly related to MeCA biosynthesis were selected from KEGG pathway analysis. Their expression was quantified in T-51 and 51-13. Total RNA was extracted from mycelia of 7 days old PDB cultures of T-51 and 51-13 by TRizol^®^ reagent (Invitrogen Corporation, Carlsbad, CA, USA). A reverse transcription reaction was performed with 1 μg of mRNA using a M-MuLV First Strand cDNA Synthesis Kit (Sangon Biotech, Shanghai, China) with Oligo dT Primer. The qRT-PCR primer pairs were designed by NCBI Primer-BLAST tool (https://www.ncbi.nlm.nih.gov/tools/primer-blast/, accessed on 18 November 2021) based on the data from transcriptome sequencing (Table S1). qRT-PCR was performed with Hieff^®^ qPCR SYBR^®^ Green Master Mix (Yeasen Biotechnology (Shanghai) Co., Ltd., Shanghai, China). *T. koningiopsis* transcriptional elongation factor gene (*Tk-tef*) was used as the internal control. qRT-PCR was independently performed three times. The relative expression of each gene was calculated using 2^−ΔΔCt^ method.

## 3. Results

### 3.1. Morphology and Genome of T-51 and 51-13

**Figure 1:**
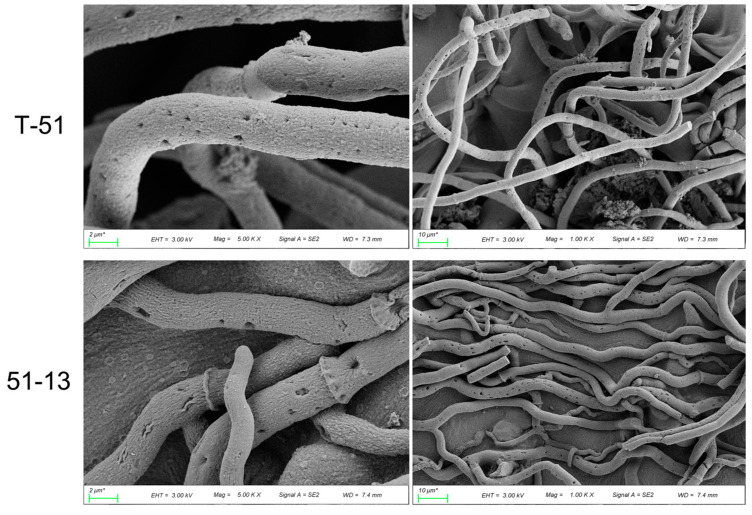
Mycelial morphology of T-51 and 51-13 observed using SEM.

SEM observation revealed that the mycelia of both T-51 and 51-13 were healthy, stout, and plump, with some holes of approximately 0.5-μm diameter. SEM did not reveal any difference between T-51 and 51-13 (Figure 1). According to the DNA sequencing data, T-51 genome length was 38.27 Mb and resequencing mapping rate of 51-13 was 98.56% (Table S2). In total, 264 SNPs and 81 indels were detected in 51-13, only 13 SNPs were in exonic area and were nonsynonymous, and 4 indels were in exonic area (Table S3). None of these genes were related to metabolites in blast. The DNA sequencing result indicates that ThHV1 and ThHV1-S exhibited little or no effect on the DNA level of the host. 

### 3.2. Antifungal Activity of the CF of T-51 and 51-13

**Figure 2:**
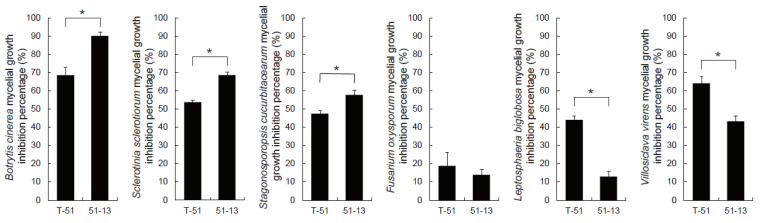
Inhibition of mycelial growth of various plant pathogenic fungi by the CF of T-51 and 51-13. * *p* < 0.05; student’s *t*-test.

**Figure 3:**
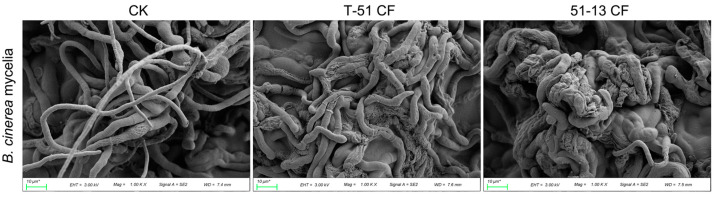
Images obtained by scanning electron microscopy showing the mycelial morphology of *B. cinerea* growing on PDA media (CK) and PDA added with the CF of T-51 or 51-13 (10% *v*/*v*).

Six species of plant pathogenic fungi including *B. cinerea*, *S. sclerotiorum*, *S. cucurbitacearum*, *F. oxysporum*, *L. biglobosa*, and *V. virens* were used for evaluating the antifungal activity of the CF of T-51 and 51-13. The antifungal activity of the CF of T-51 and 51-13 against different fungal pathogens was different. The CF of T-51 and 51-13 strongly inhibited the mycelial growth of *B. cinerea* and *S. sclerotiorum* and weakly inhibited the mycelial growth of *F. oxysporum*. The CF of 51-13 exhibited significantly stronger inhibitory effect on mycelial growth of *B. cinerea, S. sclerotiorum*, *and S. cucurbitacearum* than that of T-51 (Figure 2). However, on the mycelial growth of *L. biglobosa* and *V. virens*, the CF of T-51 CF exhibited significantly stronger inhibitory effect than that of 51-13 (Figure 2). The mycelial morphology of *B. cinerea* treated with the CF of T-51 and 51-13 was observed using an SEM. It was highly deformed compared with the untreated control. Healthy mycelia were stretched and plump. However, the mycelia treated with the CF were abnormal, exhibiting clumping, twisting, and shrinking (Figure 3). SEM observation revealed that the abnormal symptoms of *B. cinerea* were more severe after treatment with the CF of 51-13 than that of T-51 (Figure 3).

### 3.3. The Antifungal Activity of the VOCs of T-51 and 51-13

**Figure 4:**
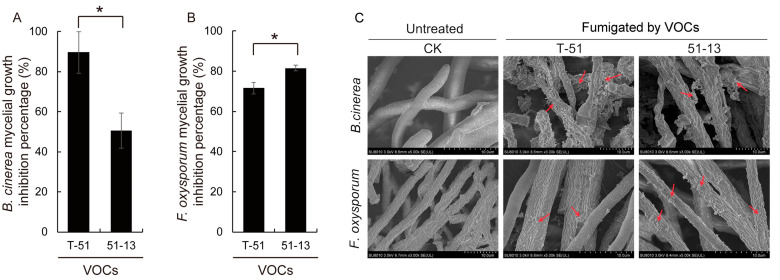
The effect of the volatile organic compounds produced by T-51 and 51-13 on the mycelial growth of *B. cinerea* (**A**) and *F. oxysporum* (**B**). Their mycelial morphology (**C**) was scanned under an SEM. * *p* < 0.05; student’s *t*-test. The red arrow showed the extracellular substance.

The mycelial growth of *B. cinerea* and *F. oxysporum* was strongly suppressed when fumigated with the VOCs of T-51 and 51-13. However, the inhibitory activity was not identical for T-51 and 51-13 (Figure 4). The VOCs of T-51 exhibited significantly stronger inhibition of *B. cinerea* than those of 51-13 (Figure 4A). However, the result was opposite in *F. oxysporum* (Figure 4B). SEM observation revealed that the mycelia of *B. cinerea* were seriously affected by the VOCs of *Trichoderma* compared with the control (Figure 4C). The mycelia in the control were smooth, plump, and strong. Similar extracellular substance was observed on the mycelia of *F. oxysporum* and the treatment with the VOCs of 51-13 exhibited more severe symptoms than that of the VOCs of T-51, which is consistent with the results given in Figure 4A,B. 

### 3.4. Comparison of Transcriptome of T-51 and 51-13

**Figure 5:**
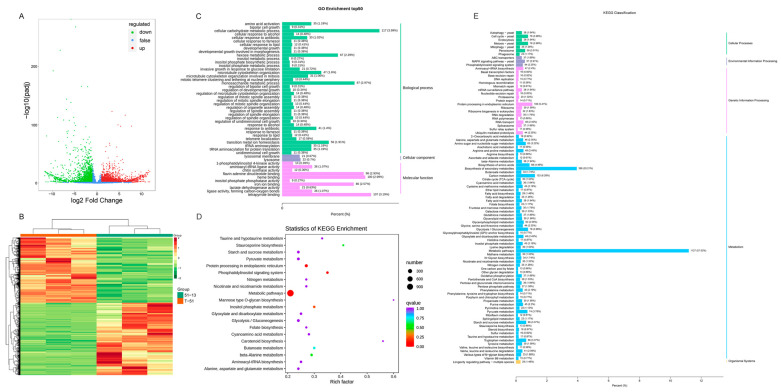
Transcriptome analysis of T-51 and 51-13. (**A**) Volcano plot of differentially expressed genes (DEGs); red and green dots represent significantly up- and downregulated genes, respectively. (**B**) Heatmap of the DEGs based on hierarchical clustering analysis. (**C**) Top 50 terms of the gene ontology (GO) enrichment of the DEGs. The percentage of the column represents the ratio of the enriched DEGs to the total annotated genes. (**D**) KEGG enrichment of the DEGs. Rich factor represents the ratio of the enriched DEGs to the total genes annotated in the corresponding pathway. (**E**) KEGG classification of the DEGs. The percentage of the column represents the ratio of the enriched DEGs to the total annotated genes.

In the RNA sequencing, we obtained 54.78 Gb clean data for 6 samples and at least 8.25 Gb data for each sample. A total of 5531 DEGs were detected between T-51 and 51-13, containing 2904 up- and 2627 downregulated genes in 51-13 (Figure 5). The DEGs were enriched by GO and KEGG enrichment analyses. In GO enrichment, 38 of the top 50 GO terms were from biological process, in which cellular carbohydrate, monosaccharide, and hexose metabolic process were the most significantly enriched between T-51 and 51-13. In the 10 molecular function terms, tetrapyrrole binding, heme binding, flavin adenine dinucleotide binding, and iron ion binding were the most significantly enriched (Figure 5C). Based on the KEGG enrichment, 1127 DEGs related to metabolic pathways and 396 DEGs related to biosynthesis of secondary metabolites were clearly enriched (Figure 5E). The KEGG enrichment results suggest that the metabolites were significantly affected by ThHV1 and ThHV1-S. 

### 3.5. Metabolic Differences between T-51 and 51-13

**Figure 6:**
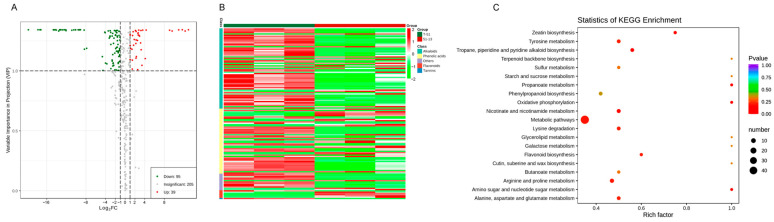
Metabolomic profiling of T-51 and 51-13. (**A**) Volcano plot of differential secondary metabolites (DSMs). Red and green dots represent the significantly up- and downregulated metabolites, respectively. (**B**) Heatmap of the DSMs based on hierarchical clustering analysis. (**C**) KEGG enrichment of the DSMs. Rich factor represents the ratio of the enriched DSMs to the total metabolites annotated in the corresponding pathway.

A total of 339 metabolites were detected, including 134 DSMs (39 up- and 95 downregulated), between T-51 and 51-13. The remaining 205 were insignificant (Figure 6A). The 134 DSMs included 63 alkaloids, 51 phenolic acids, 6 flavonoids, 1 tannin, and 13 others. The 39 upregulated DSMs included 19 phenolic acids, 12 alkaloids, 5 flavonoids, and 3 others. The DSMs were enriched by KEGG. The result indicates that the most enriched KEGG pathway is the metabolic pathway (Figure 6C).

### 3.6. Antifungal Metabolite Candidates Affected by ThHV1 and ThHV1-S

**Figure 7:**
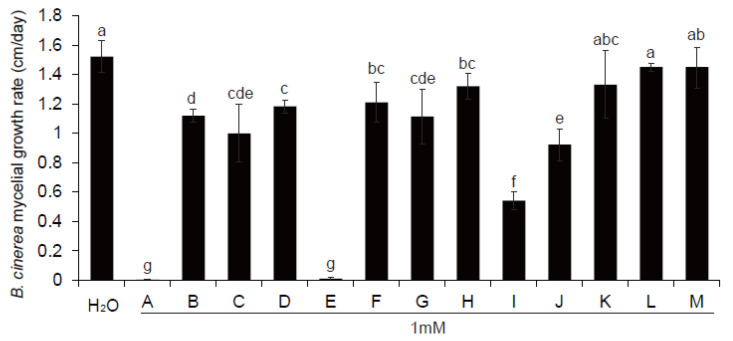
Effect of 13 compounds (A–M; Table 1; 1 mM) on the mycelial growth of *B. cinerea*. The same lowercase letters indicate that the difference was not significant at *p* < 0.05, according to Duncan’s multiple range tests.

The DSMs were filtered and the blank compounds in PDB media were removed, which may be formed by the differential nutritional consumption capacity of T-51 and 51-13. A total 27 of secondary metabolites were screened from the upregulated DSMs, containing 8 alkaloids, 11 phenolic acids, 5 flavonoids, and 3 others (Table 1). Overall, 13 compounds (1 mM; Table 1, last column) were tested for the antifungal activity. Compared with the PDA control, 10 of them significantly decreased the mycelial growth of *B. cinerea*. Indole-3-lactic acid (A) and MeCA (E) exhibited the strongest antifungal activity against *B. cinerea* as the mycelia of this pathogen hardly grew (<0.01 cm/day) on the PDA with 1 Mm of compound A or E. Moreover, 3,4,5-trimethoxycinnamic acid (I) and salicylamide (J) exhibited strong antifungal activity with the inhibition of 64.4% and 39.2%, respectively. N-acetyl-L-glutamine (B), N-acetyl-L-cysteine (C), hordenine (D), 5,6-dihydrouracil (F), hexadecanedioic acid (G), and quercetin (H) exhibited weak antifungal activity with the inhibition ranging from 13.3% to 34.3%. 2-Aminoethanesulfonic acid (K), 2-picolinic acid (L), and 2-deoxyguanosine (M) did not exhibit significant inhibitory activity compared with the control (Figure 7). 

### 3.7. Antifungal Activity of MeCA In Vitro 

**Figure 8:**
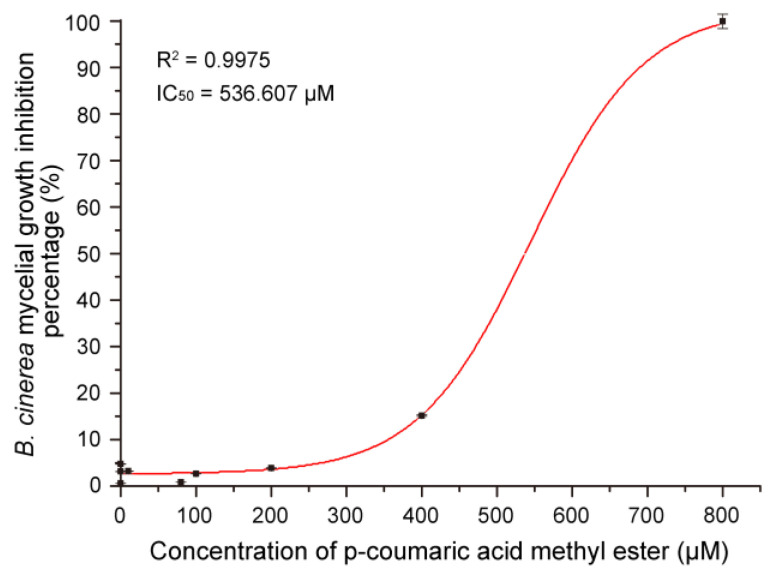
Inhibition curve of p-coumaric acid methyl ester against *B. cinerea*.

According to the bioassay results of the 13 compounds, MeCA was selected for further experiments. Various concentrations of MeCA (10 nM to 800 μM) were used to develop a dose response curve for *B. cinerea*. The IC_50_ was 536.607 μM based on the dose response curve regression equation; R^2^ of the regression equation was 0.9975 (Figure 8).

### 3.8. MeCA Related Gene Expression 

**Figure 9:**
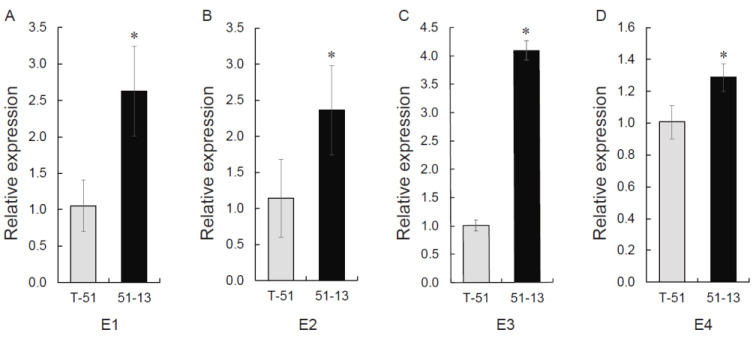
Relative quantification of four genes probably involved in the synthesis of p-coumaric acid methyl ester, which were selected by upregulation in transcriptome analysis. The data are expressed as means ± SD (n = 4). * *p* < 0.05; student’s *t*-test. (**A**) E1, (**B**) E2, (**C**) E3, (**D**) E4.

Four upregulated DEGs (E1 to E4) were selected from the transcriptome data. The KEGG annotation of E1, E2, E3, and E4 was tyrosinase, FAD dependent monooxygenase, tyrosinase, and 4-coumarate-CoA ligase, respectively (Table S1). According to the KEGG database, these genes are associated with the biosynthesis of p-coumaric acid, which is the precursor of MeCA. The relative expression of these four genes was detected for verification. The expression was quantified in T-51 and 51-13. The expression of E1, E2, E3, and E4 genes was significantly higher in 51-13 than in T-51, which was consistent with the RNA-seq results (Figure 9). This result indicates that the mycovirus affected gene expression in the fungus, resulting in the change in metabolites.

## 4. Discussion

In our previous study, we reported a mycovirus ThHV1 and its defective RNA ThHV1-S in *T. harzianum* isolate T-70D and its successful transmission into *T. koningiopsis* T-51. The derivative strain 51-13, infected by ThHV1 and ThHV1-S, exhibited stronger antifungal activity than T-51 against *B. cinerea* in vitro and in vivo [19]. In this study, the difference in antifungal activity of T-51 and 51-13 and the underlying mechanism were further studied to analyze the role of mycovirus ThHV1 and ThHV1-S in the metabolism of host.

We found that 51-13 exhibited abnormal colony morphology, slower growth, and rare sporulation compared with its parental isolate T-51 [19]. According to SEM analysis, there was no difference between the mycelia of T-51 and 51-13. Interestingly, many holes were observed on the mycelia of T-51 and 51-13, which may explain the successful transmission of the mycovirus. 

Plant pathogenic fungi from six different genera were used to evaluate the antifungal activity of T-51 and 51-13. Our previous study reported that the mycovirus did not change the biomass of T-51 when cultured in PDB [19]. However, compared with the CF of T-51, the CF of 51-13 exhibited a larger inhibitory effect against *B. cinerea*, *S. sclerotiorum*, and *S. cucurbitacearum* but a lower inhibitory effect against *L. biglobosa* and *V. virens*. Further, the VOCs of 51-13 exhibited a lower inhibitory effect against *B. cinerea* but a larger inhibitory effect against *F. oxysporum*. These results indicate that the levels of more than one antifungal metabolite were altered in the CF of 51-13 compared with that of T-51.

Mycoviruses have been reported to affect the secondary metabolism of host fungi. For example, SsHADV-1 improved the attraction of its host by a mycophagous insect *Lycoriella ingenua* via changing the production of repellent volatile substances of *S. sclerotiorum* host [4]. In *Ustilago maydis*, which causes corn smut, some strains containing a dsRNA were observed to produce killer protein to inhibit the sensitive strain [23]. A partitivirus BcPV2 altered the VOCs of its host, changing the antifungal activity [7]. The results of this study suggest that the ThHV1 and/or ThHV1-S in 51-13 may affect metabolite levels in the host. According to our previous study, other derivatized strains of T-51, (51-70-2 and 51-70-4), which were only infected by ThHV1, did not exhibit biological and mycoparasitic changes. This suggested that only ThHV1-S played the key role in changing the host phenotype [19]. Further studies are needed to assess whether the complete mycovirus ThHV1 or the defective RNA ThHV1-S alone could affect the metabolites of its host. 

The genome DNA sequencing revealed that only a few bases changed in 51-13; the difference in nucleic acid site may be due to the mutation in the strain during subculture or sequencing error. None of the SNPs and indels related to the metabolite based on gene function analysis. However, in the transcriptomic and metabolomic analyses, 5531 DEGs and 134 DSMs were detected between T-51 and 51-13, which indicated that the mycovirus has a great impact on the host at transcriptional level, causing significant phenotypic changes, including the host’s biological characteristics and metabolic processes. In this study, we inferred that the major changes in 51-13 were mainly due to the existence of the defective RNA ThHV1-S.

Higher antifungal activity of the 51-13 against *B. cinerea* suggests that upregulated DSMs should contain compounds with antifungal activity. Overall, 27 upregulated DSMs were detected in 51-13 when compared with T-51. Thirteen of these compounds were assessed for their antifungal activity. The results indicated that indole-3-lactic acid and MeCA exhibited the strongest antifungal activity against *B. cinerea*. Indole-3-lactic acid is a naturally occurring indole derivative produced by bacteria and fungi growing at various sites such as plant root [24], human digestive tract [25], and soil [26]. It was considered as a weak auxin analogue but having no antagonistic effects for plant [26]. However, it was not clear if indole-3-lactic acid can control plant pathogenic fungi. 

MeCA exhibited antifungal activity against *Cladosporium cucumerinum* [27], *A. alternata*, *Curvularia lunata*, *F. moniliforme*, *F. pallidoroseum*, and *Helminthosporium* sp. [28,29]. In this study, MeCA strongly inhibited the growth of *B. cinerea* with the IC_50_ of 536.607 μM. According to the data of Daayf et al., the IC_50_ of MeCA against *B. cinerea*, *Pythium ultimum*, and *P. aphanidermatum* were approximately 10 mg/L [30], and Yuan et al. reported that the IC_50_ of MeCA to *A. alternata* was approximately 25 mg/L [29]. The difference may be because of the difference resistance in fungal species and strains to MeCA. Four genes possibly related to the synthesis of MeCA were selected from the upregulated DEGs, and their expression was upregulated in the mycelia of 51-13 compared with those of T-51, as revealed by qRT-PCR. These results indicate that the increased antifungal activity of the CF was probably due to ThHV1 and ThHV1-S via increased MeCA production. The absolute quantification of MeCA and interaction between *Trichoderma* and its mycovirus will be explored in future. 

In summary, this study suggests that ThHV1-S, a defective RNA of ThHV1, affected the transcription of the host and affected the metabolite levels and composition, and improved the antifungal activity of its host against *B. cinerea*. With the easy transmission ability of ThHV1 and ThHV1-S, this study provides new insights into fungal engineering to improve antibiotic production. 

## Figures and Tables

**Table 1:** The compounds upregulated in the CF of 51-13 compared with that of T-51.

Formula	Compounds	CAS	Class I	Log_2_ FC	Bioassay No.
C4H6N2O2	5,6-Dihydrouracil	504-07-4	Alkaloids	12.92	F
C10H13N5O4	2′-Deoxyguanosine	961-07-9	Alkaloids	12.25	M
C16H12O7	3-O-Methylquercetin	1486-70-0	Flavonoids	10.94	-
C13H12O8	Cis-Coutaric acid	67920-37-0	Phenolic acids	9.59	-
C2H7NO3S	2-Aminoethanesulfonic acid	107-35-7	Phenolic acids	9.08	K
C12H14O5	3,4,5-Trimethoxycinnamic acid	90-50-6	Phenolic acids	4.29	I
C5H9NO3S	N-Acetyl-L-Cysteine	616-91-1	Alkaloids	4.10	C
C11H11NO4	Methyl dioxindole-3-acetate	57061-18-4	Others	3.59	-
C6H5NO2	2-Picolinic acid	98-98-6	Phenolic acids	3.43	L
C23H39NO8	Trichodermoside	1226878-09-6	Others	3.27	-
C10H15NO	Hordenine	539-15-1	Alkaloids	3.22	D
C20H20O5	2,4,2′,4′-tetrahydroxy-3′-prenylchalcone	-	Flavonoids	3.16	-
C7H7NO2	Salicylamide	65-45-2	Alkaloids	2.94	J
C7H12N2O4	N-Acetyl-L-Glutamine	2490-97-3	Alkaloids	2.83	B
C16H30O4	Hexadecanedioic acid	505-54-4	Phenolic acids	2.78	G
C10H10O3	p-Coumaric acid methyl ester	3943-97-3	Phenolic acids	2.68	E
C11H11NO3	Indole-3-lactic acid	1821-52-9	Phenolic acids	2.66	A
C17H16O6	4′,6-Dihydroxy-5,7-dimethoxyflavanone	6951-57-1	Flavonoids	2.60	-
C9H10O4	Methyl 2,4-dihydroxyphenylacetate	67828-42-6	Phenolic acids	2.52	-
C12H14N2O	Acetryptine	3551-18-6	Alkaloids	2.29	-
C22H18O9	Bis(p-Coumaroyl)malic acid	-	Phenolic acids	2.25	-
C5H10O4	2,3-Dihydroxy-3-Methylbutanoic Acid	1756-18-9	Phenolic acids	2.24	-
C21H26O6	5-Hydroxy-1,7-bis(4-hydroxy-3-methoxyphenyl)heptan-3-one	-	Others	2.21	-
C9H10O4	(S)-2-Hydroxy-3-(4-Hydroxyphenyl)Propanoic Acid	23508-35-2	Phenolic acids	2.19	-
C15H10O7	Quercetin	117-39-5	Flavonoids	2.10	H
C15H24N2O17P2	Uridine 5′-diphospho-D-glucose	133-89-1	Alkaloids	1.86	-
C15H10O6	Isoscutellarein	41440-05-5	Flavonoids	1.46	-

## Data Availability

Not applicable.

## Supplementary Materials

The following supporting information can be downloaded at: https://www.mdpi.com/article/10.3390/jof9020175/s1, Table S1: MeCA related gene in transcriptome sequencing and their primers for qRT-PCR, Table S2: Comparison of DNA sequencing of T-51 and 51-13, Table S3: SNP and indel analysis of 51-13.
