# Supplementary material for: The Effect of Trichoderma harzianum Hypovirus 1 (ThHV1) and Its Defective RNA ThHV1-S on the Antifungal Activity and Metabolome of Trichoderma koningiopsis T-51
Source: J Fungi (Basel). 2023 Jan 28;9(2):175. doi: 10.3390/jof9020175 (PMC9959424; doi:10.3390/jof9020175)
Supplement: Supplementary file 1 [file jof-09-00175-s001.zip › jof-2081474-supplementary.pdf]

Table S1. MeCA related gene in transcriptome sequencing and their primers for qRT-PCR

Primer Name		sequence (5' - 3')	PCR product size (bp)	RNA-seq gene ID	log2FC	KEGG blast
E1	Forwards	CACCGTCTTCGTCGAATGGA	168	Cluster-1420.27977	4.163989	K00505 tyrosinase [EC:1.14.18.1] | (RefSeq) Di-copper centre-containing (A)
	Reverse	GCACAGCTGATAGGCACTGA				
E2	Forwards	TTGTTCTCGATTGGCTGCCT	122	Cluster-2800.0	3.278433	K18386 FAD dependent monooxygenase [EC:1.14.13.-] | (RefSeq) FAD binding domain protein (A)
	Reverse	ACTCGCCGAGGCTGAATATG				
E3	Forwards	AAAAGCCGGCAGCTTGATTG	169	Cluster-1420.24841	2.518436	K00505 tyrosinase [EC:1.14.18.1] | (GenBank) tyrosinase 2 (A)
	Reverse	GGTTTGACCAGAGCGAGTGA				
E4	Forwards	GGTCCTGAAAATGGCCTGGA	168	Cluster-1420.10766	1.594481	K01904 4-coumarate--CoA ligase [EC:6.2.1.12] | (RefSeq) 4-coumarate:coenzyme A ligase (A)
	Reverse	GGTTGTGATCAAACGCTCCG				
Tk-tef1	Forwards	TACAAGGGCTGGGAGAAGGA	196	-	-	-
	Reverse	TACCGGGCTTGAGGATACCA				

Table S2. Comparison of DNA sequencing of T-51 and 51-13

T-51 (reference genome)	51-13 (Resequencing)			
**Total sequence length (nt)**	**Total_reads**	**Mapped_reads**	**Mapping_rate**	**Avg.Depth(X)**
38274022	13289446	13098381	98.56%	37.01

Table S3. SNP and indel analysis of 51-13

SNP		Indel	
**Type**	**Number**	**Type**	**Number**
Exonic	20	exonic	4
Synonymous	7	frameshift_deletion	1
Nonsynonymous	13	frameshift_insertion	2
Intronic	7	nonframeshift_deletion	1
Intergenic	214	intronic	5
Upstream	11	intergenic	33
Downstream	11	upstream	13
upstream;downstream	1	downstream	22
		upstream;downstream	4
**Total**	**264**	**Total**	**81**
